# Yellow Fever Vaccine-Related Neurotropic Disease in Brazil Following Immunization with 17DD

**DOI:** 10.3390/vaccines11020445

**Published:** 2023-02-15

**Authors:** Flora de Andrade Gandolfi, Cassia Fernanda Estofolete, Marcia Catelan Wakai, Andreia Francesli Negri, Michela Dias Barcelos, Nikos Vasilakis, Mauricio Lacerda Nogueira

**Affiliations:** 1 Laboratório de Pesquisas em Virologia (LPV), Faculdade de Medicina de São José do Rio Preto (FAMERP), São José do Rio Preto 15090-000, SP, Brazil; 2 Hospital da Criança e Maternidade de São José do Rio Preto, São José do Rio Preto 15091-240, SP, Brazil; 3 Hospital de Base de São José do Rio Preto, São José do Rio Preto 15090-000, SP, Brazil; 4 Municipal Health Departament, São José do Rio Preto 15084-010, SP, Brazil; 5 Department of Pathology, The University of Texas Medical Branch, Galveston, TX 77555, USA; 6 Department of Preventive Medicine and Population Health, The University of Texas Medical Branch, Galveston, TX 77555, USA; 7 Center for Vector-Borne and Zoonotic Diseases, The University of Texas Medical Branch, Galveston, TX 77555, USA; 8 Center for Biodefense and Emerging Infectious Diseases, The University of Texas Medical Branch, Galveston, TX 77555, USA; 9 Center for Tropical Diseases, The University of Texas Medical Branch, Galveston, TX 77555, USA; 10 Institute for Human Infection and Immunity, The University of Texas Medical Branch, Galveston, TX 77555, USA

**Keywords:** yellow fever, vaccine, serious adverse events, neurotropic disorder

## Abstract

The disease burden of yellow fever virus infection (YFV) is quite high in the tropics where vaccination coverage is low. To date, vaccination is the most effective control strategy to mitigate and eliminate the burden of YF disease. The licensed YF vaccines are safe and effective and serious adverse events are rare. Herein, we report three cases of neurological syndrome, compatible with meningoencephalitis following 17DD vaccination. In all cases, YFV-specific IgM antibodies were detected in the cerebrospinal fluid. Our observations confirm the development of YF vaccine-associated neurotropic disease, a rare serious adverse event, from which all three patients have fully recovered without any long-term sequelae. This report reinforces the need for awareness among health professionals to recognize and effectively manage such events in a timely manner.

## 1. Introduction

During the last decade, several yellow fever virus (YFV) outbreaks were reported in Western, Central, and Eastern Africa, and South America [1]. Clinical manifestations of infection present with a wide clinical spectrum, ranging from a mild acute febrile flu-like disease to a severe and fulminant hepatorenal syndrome with high morbidity and mortality [2,3].

Currently, there are two live attenuated yellow fever vaccines in use worldwide (17D-204 and 17DD), which both originated from the 17D vaccine developed in 1937 [3]. In 2019 and 2020, the YF vaccines supply available worldwide was 135 and 150 million doses, respectively, and will likely increase due to the Eliminate Yellow fever Epidemics (“EYE”) global initiative for eliminating the disease by 2026 [4]. Although both vaccines are safe and effective, rare serious adverse events (SAEs) have been reported over the years [5]. They are classified as (i) hypersensitive reactions, (ii) YF vaccine-associated neurological disease (YEL-AND), and (iii) YF vaccine-associated viscerotropic disease (YEL-AVD), with the latter being less frequent but potentially fatal [5,6,7].

Safe vaccination and knowledge of how to respond to public concerns have a major impact on the success or failure of immunization programs. To this end, the analysis of cases and the dissemination of quality information for the recognition of post-vaccination adverse events, especially the serious and rare ones, strengthen the pharmacovigilance process in vaccines and provides subsidies for health professionals to deal with these events. Herein, we describe three cases of YEL-AND observed in 2021 following 17-DD vaccine (Instituto de Tecnologia de Imunobiológico, Bio-Manguinhos/FioCruz, RJ, Brazil) immunization in São José do Rio Preto, São Paulo State, Brazil.

## 2. Materials and Methods

Medical History, Sample Collection, and Diagnostic Analyses

Herein, we describe three cases through retrospective analysis of clinical data (symptoms, laboratory, and radiologic observations) obtained from the medical records of meningoencephalitis-suspected pediatric patients admitted between July and August 2021 in the Hospital da Criança e Maternidade (HCM) de São José do Rio Preto, São Paulo, Brazil. Cerebrospinal fluid (CSF) samples from meningoencephalitis cases are routinely screened for a panel of arboviruses (Saint Louis encephalitis virus (SLEV); West Nile virus (WNV); Rocio virus (ROCV); Chikungunya virus (CHIKV); dengue virus (DENV); Zika virus (ZIKV); and YFV) by the Sao Paulo State reference laboratory Institute Adolfo Lutz.

According to the Brazilian Ministry of Health, the definition of YEL-AND-suspected cases, requires the presence of fever, seizures, neurological impairment associated with CSF alterations compatible to viral meningoencephalitis without other detectable causes, and within the first 30 days following yellow fever vaccination [7].

## 3. Results

### 3.1. Case 1

**Figure 1:**
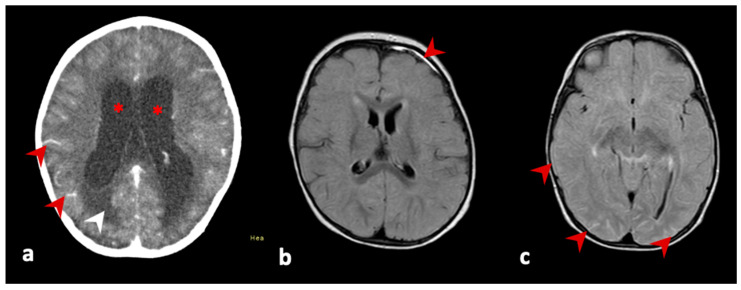
(**a**) Brain nuclear magnetic resonance (NMR) imaging of patient one. Dilatation of the ventricular system (asterisks) with signs of ependymal transudation (white arrowhead), and abnormal diffuse leptomeningeal enhancement (red arrow heads) in the image acquired with pre-contrast T1-weighted images. (**b**) Patient two’s radiologic findings. Focal pachymeningeal enhancement in the left frontal region (arrow) in the image acquired using fluid-attenuated inversion-recovery (FLAIR). (**c**) Patient three’s radiologic findings. Arrows indicate the presence of diffuse leptomeningeal enhancement in the image acquired using fluid-attenuated inversion-recovery (FLAIR).

A four-year-old female patient was admitted to the hospital on 25 August 2021 due to a month-long history of progressive symptoms: weight loss, hyporexia, emesis, and evolving with bradycardia and bradypnea. Brain computed tomography (CT) revealed signs of hypertensive hydrocephaly, and an external ventricular drainage was performed immediately thereafter. Brain nuclear magnetic resonance (NMR) imaging performed on the next day suggested an inflammatory-infectious process and a cerebral biopsy showed an area of granulomatous chronic inflammatory process with focal necrosis and histiocyte infiltration (Figure 1a). Empirical treatment for bacterial, fungal, and viral meningitis were initiated. Due to the chronicity of the symptoms, the NMR, and biopsy findings with granulomatous characteristics, empirical treatment for neurological tuberculosis (TB) was also initiated. For that hypothesis, ceftriaxone 50 mg/kg/dose twice daily, fluconazole 12 mg/kg/day, and rifampicin 225 mg + isoniazid 150 mg + pyrazinamide 450 mg once daily were administered. A blood examination showed normal liver, hematological, and kidney functions.

Blood and CSF samples were submitted for screening against a comprehensive panel of infectious agents (Table 1). No agents were identified in serum, and CSF screening showed leukocytes 17 cells/mm^3^, lymphocyte predominance (86%), protein 153 mg/dL, glucose 36 mg/dL, and bacteria cultures and RT PCR for TB were negative. Enzyme-linked immunosorbent assays (ELISA) in CSF for anti-dengue virus (DENV), anti-Zika virus (ZIKV), anti-Saint Louis encephalitis virus (SLEV), anti-West Nile virus (WNV), anti-Rocio virus (ROCV), and anti-Chikungunya virus (CHIKV) IgM were all negative, except for anti-YFV IgM.

The patient had received the second dose of the 17DD vaccine 36 days prior to the onset of symptoms and was hospitalized for 58 days, of which 24 days were spent in the intensive care unit (ICU). The external ventricular drainage was removed on day 21 post hospital admission. A secondary brain NMR showed significant improvement, and the patient was discharged on 22 October 2021. On follow-up 21 days after discharge, the patient showed no signs of symptom recurrence or any evidence of sequelae.

### 3.2. Case 2

A 10-month-old female was admitted on 7 July 2021 with a history of fever, vomiting for 6 days, progressive somnolence, lack of responsiveness, tonic–clonic seizures, and left hemiparesis. She had received the first 17DD dose 20 days prior to the onset of symptoms at 9 months and 7 days of age. The CSF analysis showed leukocytes 150 cells/mm^3^ with a predominance of lymphocytes (72%), protein 110 mg/dl, and glucose 45 mg/dl. Culture for bacteria was negative. The patient was admitted to ICU, and a 50 mg/kg/dose of ceftriaxone twice daily and a 20 mg/kg/dose of acyclovir treatment thrice daily was initiated for probable bacterial and viral meningoencephalitis. Brain NMR imaging identified focal pachymeningeal enhancement in the frontal brain area. The electroencephalogram (EEG) showed bilateral degree I slowing, without epileptiform disorder (Figure 1b). Symptoms were controlled and cleared following treatment, and the patient was discharged seven days following hospital admission. Subsequent to her discharge, CSF screening with a comprehensive ELISA panel for arboviruses, showed positive for anti-YFV IgM (Table 1). On clinical follow up 29 days after discharge from the hospital, the patient did not exhibit any recurring symptoms or sequelae.

### 3.3. Case 3

A 9-month-old female was admitted to the hospital on 6 August 2021 because of a week-long history of fever, hyporexia, and adynamia; a history of diarrhea, emesis, lethargy, and irritability whose onset presented fourteen days following the first dose of the 17DD vaccine. On admission, the patient received intravenous antibiotics (a ceftriaxone 50 mg/kg/dose bid and oxacillin 50 mg/kg/dose bid), and blood and CSF samples were collected. Analysis of CSF showed an increased leukocyte count (70 cells/mm^3^ with lymphocytes 73% and monocytes 25%), protein 35 mg/dL, and glucose 51 mg/dL. Culture for bacteria was negative, anti-dengue IgM was negative, and anti-YFV IgM was positive using ELISA. In addition, brain NMR imaging indicated diffuse leptomeningeal enhancement (Figure 1c). The detailed clinical data are shown in Table 1. The patient remained hospitalized for 6 days without the need for admission to the ICU and was discharged. On clinical follow-up 53 days after discharge, the patient did not exhibit any recurring symptoms or sequelae.

## 4. Discussion

The three reported cases of yellow fever vaccine-associated neurotropic disease (YEL-AND) following 17DD vaccination were detected by our health surveillance system. Although, the three events occurred in a short period, any relationship between the vaccine batch or vaccine administration establishment was not identified. Yet, none of the patients had a medical condition prior to the event or diagnosed during the follow-up. These case reports represent a rare phenomenon, albeit quite significant for public health safety.

Historically, the two live attenuated virus vaccines were developed in the 1930s to control YF disease. The 17D vaccine was developed in 1937 following 176 passages of the wild-type strain Asibi in mouse and chicken derived cell lines, and shortly thereafter the French neurotropic vaccine (FNV) was developed from a YFV strain isolated at the Institute Pasteur in Dakar (reviewed in [8]), and although effective, it was discontinued in the early 1980′s due to the high rate of post-vaccination complications [9]. Currently, only 17D-204 and 17DD are used around the world [3,8]. The administered 17DD vaccine is manufactured in Brazil by Bio-Manguinhos/Fiocruz, and it has been routinely used in several South American countries, while the 17D-204 vaccine is used outside Brazil and manufactured by Sanofi-Pasteur (France), Pasteur Institute Dakar (Senegal) and Federal State Budgetary Scientific Institution (Russia) [6,10]. Both are considered safe and effective, despite very rare cases of serious adverse events (SAEs) [8] commonly manifested as disorders affecting the immune response and the nervous system [11,12,13,14,15]. The risk of SAEs following immunization is higher in pregnant woman, thymus disorders, infants (6–8 months of age), or persons over 60 years of age [11,12,13,14,15]. Due to these risks, the Brazilian National Immunization Program applies the first dose of 17DD in infants at nine months of age, with a booster at four years of age. All three children described in this study received their first dose of the vaccine after reaching 9 months of age.

The development of YEL-AND may involve different mechanisms. For example, direct virus invasion may be responsible in meningoencephalitis syndromes, whereas in other neurological syndromes, such as Guillain–Barré syndrome and acute disseminated encephalomyelitis (ADEM), antibody and T-cell triggered autoimmunity may be involved [6]. The Brazilian Ministry of Health defines as a confirmed case of YEL-AND the presence of the following criteria: (a) symptom onset between one and 30 days after YF vaccination; (b) clinical presentation compatible with neurotropic disease; (c) CSF indicative of viral meningoencephalitis; (d) the exclusion of other causes; and (e) laboratory detection in CSF of the YFV vaccine by viral isolation, amplification, or by the presence of the anti-YFV IgM antibody [7]. Considering that the YFV IgM antibody does not cross the blood–brain barrier due to its high molecular weight, its presence in CSF may be considered as locally produced and indicative of CNS infection [14,16]. Beyond these criteria, the diagnosis of YF vaccine-related meningoencephalitis may be corroborated by complementary exams, such as neuroimaging.

In two cases presented here, the onset of symptoms occurred within the 30-day period following YF vaccination and were in line with the Brazilian Ministry of Health criteria for the definition of YEL-AND. In case 1, the onset of symptoms was mild and progressive around 30 days after vaccination, making it difficult to characterize the exact onset of symptoms. Additionally, all cases showed clinical symptoms of neurological syndromes compatible with meningoencephalitis (pleocytosis and increased protein level in CSF). YFV was the only possible cause confirmed by the presence of anti-YFV IgM antibodies.

We also carried out a short review using the main databases (PubMed, Lilacs, and Medline) using the keywords “YELLOW FEVER” AND “NEUROTROPIC” or “YELLOW FEVER VACCINE” AND ”NEUROLOGICAL” AND ” ADVERSE EVENT”, resulting in 24 articles reporting several cases of YEL-AND summarized in Table A1 [11,14,15,16,17,18,19,20,21,22,23,24,25,26,27,28,29,30,31,32,33,34,35,36]. The data showed that adults and the elderly had a higher risk for development of SAEs, and the highest occurrence of SAEs were associated with the 17D-204 vaccine and presented after administration of the first dose [11,15,16]. In contrast, our cases were associated with administration of the 17DD vaccine, and in one of the cases SAEs developed following administration of the second dose of the vaccine.

Overall, YEL-related SAE reported rates are variable. Lindsey et al., 2016 [15], using historical data from the U.S. Vaccine Adverse Event Reporting System (VAERS) database from 2007 to 2013, reported a YEL-AND rate of 0.8/100,000 YF vaccinations (17D-204 (YF-VAX^®^), Sanofi Pasteur-Swiftwater, Pennsylvania). In parts of Africa, from 2007 to 2010, the reported incidence rate of YEL-AND was 0.016/100,000 doses of 17D-204 administered [18]. Lastly, in Brazil, between 2000 and 2015, the overall reported incidence rate of YEL-AND was 0.3/100,000 doses (17DD) administered, whereas in the period between 2007–2012, the rate was 0.17/100,000 doses administered [7]. It is so important to recognize its occurrence and understand its scale, especially for building a healthcare system able to identify and treat such cases. Moreover, a close examination of such cases reported in the literature and official bulletins might mitigate inappropriate notions about vaccine safety. 

## 5. Conclusions

It is very important to emphasize that despite SAEs, the YF vaccine is safe, effective, and strongly recommended in risk areas with its benefits outweighing its risks. Moreover, the lethality from YFV infection is higher than the rates of SAEs triggered by vaccination. However, serious, and potentially fatal adverse events have been reported over the years, even though they are very rare. Thereby, it is imperative that healthcare personnel be prepared to identify such events, manage them early, and be able to critically and effectively communicate and educate the population of the benefits and the potential risks of vaccination in order to prevent misinformation and mitigate fears that may lead to reduced vaccination rates which could fuel future YFV outbreaks.

## Figures and Tables

**Table 1:** Clinical data from the patients with yellow fever vaccine-related neurological disease detected in São José do Rio Preto, São Paulo, Brazil in 2021.

ID	Gender, Age	YFV Vaccine Date, Dose	Date of Hospitalization	Time between Vaccination and Symptoms Onset	Signs and Symptoms	CSF Observations	Radiologic Observations	Other Observations	Diagnostic Tests
1;	female, 4 years of age	20 July 2021, second dose	25 August 2021	36 days	vomiting, hyporexia, non-responsiveness, weight loss of 3 kg, bradypnea, bradycardia. outcome: follow-up on 12 November 2021, no recurrence of signs and symptoms, without sequelae	leukocytes 17 cells/mm^3^, lymphocytes 86%, neutrophils 0%, protein 153 mg/dL, glucose 37 mg/dL, culture negative	brain CT: signs of hypertensive hydrocephaly (supra and infratentorial). brain NMR: increased dimensions of the ventricular system, with ependymal transudation and interstitial edema, in addition to hyperdynamic flow within the third and fourth ventricle and mesencephalic aqueduct. Note leptomeningeal enhancement in the base cysteines and cranial pairs. brain biopsy: granulomatous chronic inflammatory process with focal necrosis and histiocytes	plasma/serum: Ht 33.2%; Hb 11.8 g/dL; plat 304,000/mm^3^; leuko: 8810/mm^3^; 24.9% neutro; TB: 0.22 mg/dL; DB: 0.1 mg/dL, Cr: 0.4 mg/dL; AST: 21 UI/L; ALT: 11 UI/L; GGT: 10 UI/L; AP: 112 UI/L; INR 1.17; anti-CMV IgM negative and IgG positive; PCR for CMV: negative; anti-HIV I/II: negative; anti-Rubella IgM negative and IgG positive; FTA-abs negative; anti-toxoplasmosis IgM negative; anti-measles IgM negative; anti-EBV IgM negative; anti-HSV I/II IgM negative CSF: real-time PCR TB negative; anti-dengue IgM negative; anti-Zika IgM negative; anti-SLEV IgM negative; anti-WNV negative; anti-ROCV IgM negative; anti-CHIKV IgM negative	anti-YFV IgM positive in CSF
2;	female, 10 months of age	17 June 2021, first dose	7 July 2021	20 days	vomiting, somnolence, responsiveness, tonic-clonic seizures, fever, and left hemiparesis. outcome: follow-up on 12 August 2021, patient with no recurrence of signs and symptoms, without sequelae	leukocytes 150 cells/mm^3^, lymphocytes 72%, neutrophils 4%, protein 110 mg/dL, glucose 45 mg/dL, culture negative	EEG: bilateral degree I slowing, without non-epileptiform disorder brain NMR: focal pachymeningeal enhancement in the frontal brain area	plasma/serum: Ht 28.2%; Hb 10.1 g/dl; plat 244,000/mm^3^; leuko: 6950/mm^3^; 35.6% neutro; TB: 0.15 mg/dL; DB: 0.08 mg/dL, Cr: 0.3 mg/dL; AST: 30 UI/L; ALT: 15 UI/L; GGT: 16 UI/L; AP: 164 UI/L; INR 1.27; anti-dengue IgM negative CSF: anti-dengue IgM negative; anti-Zika IgM negative; anti-SLEV IgM negative; anti-WNV IgM negative; anti-ROCV IgM negative; RT-PCR CHIKV negative; RT-PCR DENV negative; RT-PCR SLEV negative; RT-PCR YFV negative; RT-PCR WNV negative; RT-PCR ZIKV negative	anti-YFV IgM positive in CSF
3.	female, 9 months of age	23 July 2021, first dose	6 August 2021	14 days	fever, hyporexia, adynamia, emesis, lethargy, diarrhea, irritability. outcome: follow-up on 4 October 2021, patient without sequelae	leukocytes 70 cells/mm^3^, lymphocytes 73%, monocytes 25%, protein 35 mg/dL, glucose 51 mg/dL, culture negative	brain NMR: diffuse leptomeningeal enhancement	plasma/serum: Ht 39.1%; Hb 13g/dL; plat 499,000/mm^3^; leuko: 20,160/mm^3^; 71.8% neutro; TB: 0.17 mg/dL; DB: 0.01 mg/dL, Cr: 0.5 mg/dl; ALT: 11 UI/L; INR 1.20 CSF: anti-dengue IgM negative	anti-YFV IgM positive in CSF

CSF: cerebrospinal fluid. Reference values: leukocytes ≤ 3 cells/mm^3^, lymphocytes ≥ 90%, neutrophils ≤ 10%, protein ≤ 40 mg/dL, glucose ≥ 2/3 serum level, and culture negative. Ht: hematocrit; Hb: hemoglobin; plat: platelets; leuko: leukocytes; neutro: neutrophiles; TB: total bilirubin; DB: direct bilirubin; Cr: creatine; AST: aspartate aminotransferase; ALT: alanine aminotransferase; GGT: gamma-glutamyl transferase; INR: international normalized ratio; real-time PCR TB: real time polymerase chain reaction for tuberculosis; SLEV: Saint Louis encephalitis virus; WNV: West Nile virus; ROCV: Rocio virus; CHIKV: Chikungunya virus; DENV: dengue virus; ZIKV: Zika virus; YFV: yellow fever virus; EBV: Epstein–Barr virus; HSV: herpes simplex virus; CMV: cytomegalovirus; PCR: polymerase chain reaction; FTA-abs: fluorescent treponemal antibody absorption test; CT: computed tomography; NMR: nuclear magnetic resonance; EEG: electroencephalogram.

## Data Availability

No datasets were generated or analyzed during the current study.

## Appendix A

**Table A1:** Cases of Yellow Fever vaccine-related neurological disease reported in main databases.

Reference ID	Gender, Age	Number of Cases, Clinical Presentation	Clinical Outcome	Yellow Fever Vaccine	Country, Year of Occurrence
[11]	both,16–71 years of age	10, meningitis, encephalitis, Sd Guillain–Barrè Syndrome	recovered	17D-204	USA, 1990–2002
[14]	male, 9 months of age	1, meningitis	recovered	not reported	Brazil, not reported
[15]	both, 17–76 years of age	17, encephalitis, Guillain–Barrè syndrome, aseptic meningitis, acute disseminated encephalomyelitis and transverse myelitis	recovered	17D-204	USA, 2007–2013
[16]	male, 53–63 years of age	2, transverse myelitis and cerebelar syndrome	recovered	17D	Argentina, 2008
[17]	male, 39 years of age.	1, meningitis	recovered	17D-204	Poland, 2015
[18]	male/female, not reported	6, transverse myelitis, encephalitis, aseptic meningitis, facial paralysis,	1 death and 5 recovered	17D and 17DD	Benin, Burkina Faso, Cameroon, Guinea, Liberia, Mali, Senegal, Sierra Leone, and Togo, 2007-10
[19]	male, 37 years of age	1, intermediate and anterior uveitis	recovered	17D-204	France, 2018
[20]	both, 0–85 years of age.	67, Guillain–Barré syndrome, acute disseminated encephalomyelitis cases, transverse myelitis, bilateral optic neuritis, meningoencephalitis, polyradiculoneuritis, Kinsbourne syndrome	recovered	17DD	Brazil, 2007–12
[21]	female, 23 years of age.	1, encephalitis (and viscerotropic disease)	recovered	17D-204	Brazil, 2006
[22]	male, 70 years of age	1, stroke-like syndrome	recovered	not reported	Portugal, not reported
[23]	male, 4 years of age	1, meningoencephalitis	recovered	17D-204	France, not reported
[24]	both, 21–55 years of age	4, meningitis and meningoencephalitis	recovered	17D	France, 2000–2008
[25]	female, 14–42 years of age	2, encephalitis and cerebelar syndrome	recovered	17DD	Brazil, 2007–2008
[26]	male, 56 years of age	1, longitudinal myelitis	recovered	17D-204	Argentina, 2009
[27]	both, 19–59 years of age	2, meningoencephalitis	recovered	17D	France, not reported
[28]	male, 16–71 years of age	4, meningitis and white matter disease	recovered	not reported	USA, 2001–2002
[29]	male, 56 years of age	1, meningitis	recovered	17D-204	Belgium, 2018
[30]	male, 56 years of age	1, meningoencephalitis	recovered	17D-204	Germany, not reported
[31]	male, 76 years of age	1, encephalitis (and viscerotropic disease)	recovered	17D-204	USA, 1998
[32]	female, 3 years of age	1, encephalitis	death	17D	USA, 1965
[33]	female, 67 years of age	1, meningoencephalitis and variant Creutzfeldt Jakob disease	recovered	17D-204	USA, not reported
[34]	both genders, 17–68 years of age	15, encephalitis, Guillain–Barrè syndrome, acute disseminated encephalomyelitis	recovered	17D-204	USA, 1990–2005
[35]	both genders, 0–70 years of age	12, not reported	not reported	17D-204	USA, 2000–2006
[36]	not reported	4, encephalitis, Guillain–Barrè syndrome, bulbar palsy	not reported	17D-204	not reported, 1991–2001
